# Activity‐based proteomics reveals nine target proteases for the recombinant protein‐stabilizing inhibitor *Sl* CYS8 in *Nicotiana benthamiana*

**DOI:** 10.1111/pbi.13092

**Published:** 2019-03-14

**Authors:** Philippe V. Jutras, Friederike Grosse‐Holz, Farnusch Kaschani, Markus Kaiser, Dominique Michaud, Renier A.L. van der Hoorn

**Affiliations:** ^1^ Department of Plant Sciences Plant Chemetics Laboratory University of Oxford Oxford UK; ^2^ Chemische Biologie Zentrum für Medizinische Biotechnologie Fakultät für Biologie Universität Duisburg‐Essen Essen Germany; ^3^ Centre de recherche et d'innovation sur les végétaux Université Laval Québec Canada

**Keywords:** activity‐based protein profiling, protease inhibitor, cystatin, *Sl* CYS8, *Nicotiana benthamiana*, proteomics, papain‐like cysteine proteases

## Abstract

Co‐expression of protease inhibitors like the tomato cystatin *Sl* CYS8 is useful to increase recombinant protein production in plants, but key proteases involved in protein proteolysis are still unknown. Here, we performed activity‐based protein profiling to identify proteases that are inhibited by *Sl* CYS8 in agroinfiltrated *Nicotiana benthamiana*. We discovered that *Sl* CYS8 selectively suppresses papain‐like cysteine protease (PLCP) activity in both apoplastic fluids and total leaf extracts, while not affecting vacuolar‐processing enzyme and serine hydrolase activity. A robust concentration‐dependent inhibition of PLCPs occurred *in vitro* when purified *Sl* CYS8 was added to leaf extracts, indicating direct cystatin–PLCP interactions. Activity‐based proteomics revealed that nine different Cathepsin‐L/‐F‐like PLCPs are strongly inhibited by *Sl* CYS8 in leaves. By contrast, the activity of five other Cathepsin‐B/‐H‐like PLCPs, as well as 87 Ser hydrolases, was unaffected by *Sl* CYS8. *Sl* CYS8 expression prevented protein degradation by inhibiting intermediate and mature isoforms of granulin‐containing proteases from the Resistant‐to‐Desiccation‐21 (RD21) PLCP subfamily. Our data underline the key role of endogenous PLCPs on recombinant protein degradation and reveal candidate proteases for depletion strategies.

## Introduction

Plant cells are increasingly used as alternative expression hosts for the production of recombinant proteins (Daniell *et al*., 2015; Lomonossoff and D'Aoust, 2016; Ma *et al*., 2015). However, protein proteolytic processing leads to either partial or complete degradation of recombinant proteins (Mandal *et al*., 2016; Pillay *et al*., 2014). Hundreds of genes code for proteolytic enzymes of diverse classes involved in many physiological processes in plants (Grosse‐Holz *et al*., 2018a; van der Hoorn, 2008). Controlling plant protease activity is a worthwhile approach to improve accumulation of recombinant proteins (Benchabane *et al*., 2008; Mandal *et al*., 2016). Unintended proteolysis has been mitigated in plant‐based expression systems by (i) targeting proteins to alternative cellular compartments (Benchabane *et al*., 2009); (ii) the addition of translational fusion partners to increase protein stability (Sainsbury *et al*., 2013); or (iii) the removal of protein domains and sequences targeted by endogenous plant proteases (Hehle *et al*., 2016; Zischewski *et al*., 2015). In addition, protease activity‐depleted environments have been created using gene silencing with RNAi strategies (Duwadi *et al*., 2015; Mandal *et al*., 2014) and inhibition of proteases using ectopic inhibitors in stable or transient plant expression systems (Goulet *et al*., 2010; Komarnytsky *et al*., 2006; Robert *et al*., 2013). Yet proteolytic degradation remains a major problem in the exploitation of plants as biofactories.

Although plants have diverse protease families, cysteine (Cys) proteases are a major constituent of the recombinant protein degradation machinery (Jutras *et al*., 2016; Niemer *et al*., 2014). Papain‐like cysteine proteases (PLCPs, C1A family) can be inhibited by cystatins (I12 family). Cystatins harbour a conserved motif Gln‐Xaa‐Val‐Xaa‐Gly (QxVxG) in the central region of the polypeptide chain and are competitive protease inhibitors that have strong affinity for the active site of target proteases (Benchabane *et al*., 2010; Martínez *et al*., 2012). Transgenic tobacco plants constitutively expressing a rice cystatin (OC‐I) have reduced protease activity, correlated with a higher accumulation of recombinant proteins (Pillay *et al*., 2012). Transient expression of tomato cystatin *Sl*CYS8 (Girard *et al*., 2007) in the cell secretory pathway of agroinfiltrated *Nicotiana benthamiana* leaves also prevents degradation of recombinant proteins, notably leading to higher yields of fully assembled human antibodies (Grosse‐Holz *et al*., 2018b; Jutras *et al*., 2016; Robert *et al*., 2013). An inactive version of *Sl*CYS8, which bears a proline (P) instead of a glutamine (Q) in the inhibitory loop QxVxG motif (^Q47P^ *Sl*CYS8)**,** has no stabilizing effect on recombinant proteins (Grosse‐Holz *et al*., 2018b; Jutras *et al*., 2016; Sainsbury *et al*., 2013), indicating that *Sl*CYS8 stabilizes recombinant proteins through inhibiting proteases. However, target proteases of *Sl*CYS8 and other inhibitors have not been identified to date. A better understanding of which plant proteases are inhibited by *Sl*CYS8 is needed to identify proteases that degrade recombinant proteins in molecular pharming.

Our goal was to identify the proteases that are suppressed by *Sl*CYS8 *in planta*. In tomato, the *Sl*CYS8 encoding gene is induced by jasmonic acid. *Sl*CYS8 is the eighth domain of a multicystatin that accumulates in the cytosol of leaf cells upon herbivory challenge, presumably to inhibit digestive cysteine proteases in the midgut of herbivorous arthropods (Girard *et al*., 2007). In this study, we characterized the impact of *Sl*CYS8 expression on protease activity profiles in agroinfiltrated leaves of *N. benthamiana*. We performed activity‐based protein profiling (ABPP) to determine the inhibitory spectrum of *Sl*CYS8 and proteomics to identify target proteases. ABPP is increasingly used in plant science to monitor the activity of hundreds of proteins using tagged chemical probes that react covalently and irreversibly with the active site of proteins (Morimoto and van der Hoorn, 2016). Here, we used ABPP to show that *Sl*CYS8 selectively inhibits nine papain‐like cysteine proteases in *N. benthamiana*. These proteases are likely involved in recombinant protein degradation.

## Results and discussion

### *Sl*CYS8 inhibits papain‐like cysteine proteases in the apoplast

Most recombinant proteins are targeted to the secretory pathway for post‐translational modifications and are secreted in the apoplast. Recently, peptides corresponding to 196 proteases have been identified in the extracellular space of agroinfiltrated *N. benthamiana* leaves (Grosse‐Holz *et al*., 2018a). We thus focused on extracellular proteases as protein degradation in the apoplastic space limits the accumulation of recombinant proteins (Hehle *et al*., 2011). Apoplastic fluids were isolated from plants agroinfiltrated with a *Sl*CYS8 transgene‐harbouring vector, or with an ‘empty’ vector. As negative control, we expressed the inactive version of the inhibitor, ^Q47P^ *Sl*CYS8 (Sainsbury *et al*., 2013).

**Figure 1:**
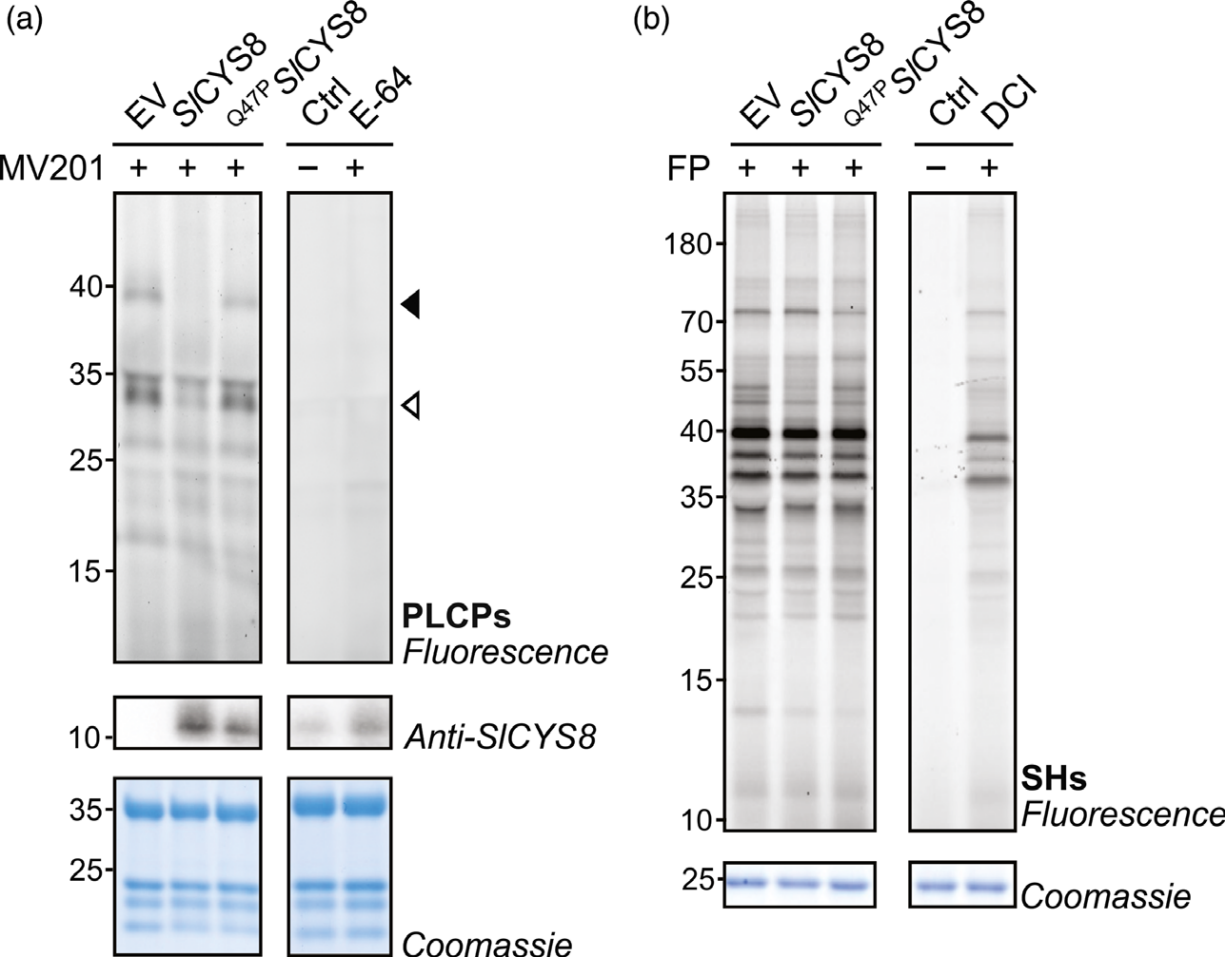
*Sl* CYS8 inhibits papain‐like cysteine proteases (PLCPs) in the apoplast of *N. benthamiana* leaves. Apoplastic fluids from plants agroinfiltrated with an empty vector control (EV) or transiently expressing *Sl* CYS8 or its inactive mutant ^Q47^ ^P^ *S* *l* CYS8 were isolated and incubated with MV201 or fluorophosphonate (FP)‐TAMRA probes to target (a) papain‐like cysteine proteases (PLCPs) and (b) Ser hydrolases (SHs), respectively. Labelled proteins were detected by in‐gel fluorescence scanning. Proteins were electrotransferred for immunodetection of *Sl* CYS8 in the apoplast. Arrows show signals with different fluorescence intensities in plants expressing *Sl* CYS8. As controls, apoplastic fluids were mixed and pre‐incubated with specific chemical inhibitors (E‐64 for PLCPs and DCI for SHs) before incubation with or without (Ctrl) the probes. Coomassie blue‐stained gels are shown as loading controls.

Activity‐based protein profiling (ABPP) was first conducted to assess whether the expression of a secreted version of *Sl*CYS8 could alter papain‐like cysteine protease (PLCP) activity in the extracellular space of *N. benthamiana* leaves (Sainsbury *et al*., 2013). PLCP activity was characterized by incubating apoplastic fluids with MV201, a fluorescent derivative of the chemical PLCP inhibitor E‐64 which has been frequently used in plants (Richau *et al*., 2012). MV201 carries a bodipy fluorophore and targets PLCPs by carrying P2 = Leu. Labelled proteases are detected and quantified using protein in‐gel fluorescent scanning. SDS‐PAGE gel analysis showed a significant reduction of PLCP activity in leaves expressing *Sl*CYS8 when compared to the empty vector control (Figure 1). The main fluorescent signals detected at ~38 and ~33 kDa are significantly decreased in the apoplast from leaves expressing *Sl*CYS8 (arrows, Figure 1a), indicating that PLCP activity was depleted. Strong signals at ~33 kDa (open arrow, Figure 1a) are likely caused by mature isoforms of various PLCP subfamilies, including RD21‐like proteases and Cathepsin B, as those proteases are active in the extracellular space of *N. benthamiana* leaves (Grosse‐Holz *et al*., 2018a). No significant effect on fluorescence intensity was detected in apoplastic fluids from plants expressing the inactive ^Q47P^ *Sl*CYS8 mutant (Figure 1a), confirming that the effect of *Sl*CYS8 is related to protease inhibition. Pre‐incubation with the PLCP inhibitor E‐64 suppressed MV201 labelling (Figure 1a), thus confirming that the detected band signals originate from PLCPs.

To determine the specificity of *Sl*CYS8 and to study potential indirect regulation of proteases by *Sl*CYS8, ABPP was performed on apoplastic fluids with an activity‐based fluorescent probe targeting Ser hydrolases. Ser hydrolases, which include subtilases (S8 family) and Ser carboxypeptidases (S10 family), are present in the extracellular space of agroinfiltrated *N. benthamiana* leaves and are involved in proteolytic processing of recombinant proteins (Grosse‐Holz *et al*., 2018a; Komarnytsky *et al*., 2006). Ser hydrolases were labelled with a fluorophosphonate (FP)‐TAMRA probe, which labels Ser residues in the active site of Ser hydrolases (Kaschani *et al*., 2009; Liu *et al*., 1999). Gel fluorescence scanning of labelled apoplastic fluids showed that activity profiles of Ser hydrolases were unchanged upon the expression of *Sl*CYS8 or the inactive ^Q47P^ *Sl*CYS8 mutant, confirming a specificity of *Sl*CYS8 to PLCPs and indicating that these Ser proteases are not regulated by *Sl*CYS8‐sensitive PLCPs (Figure 1b). These data show the alteration of extracellular PLCPs upon *Sl*CYS8 expression and identification of PLCPs as potential proteases involved in apoplastic protein degradation.

### *Sl*CYS8 targets papain‐like cysteine proteases in total leaf extracts

**Figure 2:**
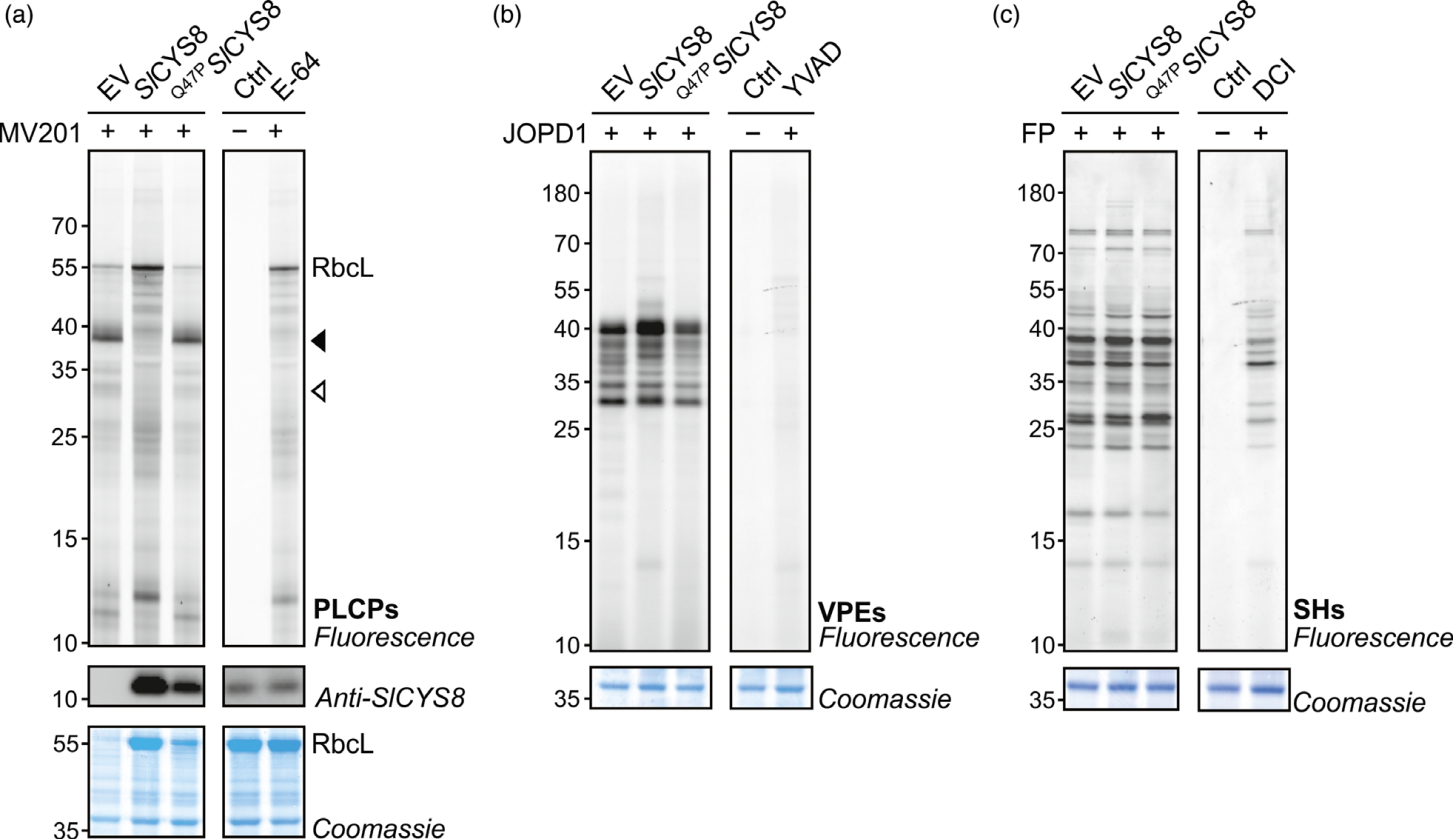
PLCPs are suppressed upon *Sl* CYS8 expression in total leaf extracts. Activity profiles of PLCPs (a), vacuolar‐processing enzymes (b) and Ser hydrolases (c) displayed by labelling with MV201, JOPD1 and FP‐TAMRA, respectively. Plants were agroinfiltrated with an empty vector control (EV) or *Sl* CYS8 or its inactive mutant ^Q47^ ^P^ *S* *l* CYS8. Total leaf extracts isolated at 6 dpi were labelled with respective probes and fluorescent‐labelled proteins were detected by in‐gel fluorescence scanning. Arrows show signals with different fluorescence intensities in plants expressing *Sl* CYS8. The large subunit of ribulose‐1,5‐bisphosphate carboxylase/oxygenase (RbcL) is stabilized during labelling in *Sl* CYS8‐expressing leaves. Proteins were electrotransferred for immunodetection of *Sl* CYS8. As controls, leaf extracts were mixed and pre‐incubated with specific chemical inhibitors (E‐64 for PLCPs, YVAD for VPEs and DCI for SHs) before incubation with or without (Ctrl) the probes. Coomassie blue‐stained gels are shown as loading controls.

Although many proteases enter the endomembrane system, not all of them accumulate in the apoplast. Some accumulate in the secretory pathway or in the vacuole and are potentially involved in proteolytic processing of recombinant proteins. To assess how *N. benthamiana* intracellular proteases respond to *Sl*CYS8 expression, total soluble proteins were extracted from plants transiently expressing *Sl*CYS8 or the inactive ^Q47P^ *Sl*CYS8 mutant. Leaf extracts were incubated with fluorescent MV201 probe to monitor PLCP activity. In contrast to apoplastic fluids, granulin‐containing proteases dominate PLCP activity in total extracts, causing strong signals at ~38 kDa (closed arrow, Figure 2a). Fluorescence intensity of these signals drastically decreased in the presence of *Sl*CYS8 (closed arrow, Figure 2a). Similar to the apoplast, the signal at ~33 kDa also decreased in leaves expressing *Sl*CYS8 (open arrow, Figure 2a). *Sl*CYS8 expression prevents the degradation of the large subunit of ribulose‐1,5‐bisphosphate carboxylase/oxygenase (RbcL) and other proteins in total extracts during labelling, causing fluorescent signals that cannot be competed by E‐64, indicating that these fluorescent signals are caused by non‐specific labelling (Figure 2a). The inactive ^Q47P^ *Sl*CYS8 mutant did not affect labelling or RbcL degradation. Taken together, these results confirm a strong inhibition of endogenous PLCP activity, both inside and outside the cells, upon *Sl*CYS8 expression in *N. benthamiana* leaves.

Legumain‐like Cys proteases (C13 family), or vacuolar‐processing enzymes (VPEs), are a group of Asn/Asp‐specific proteinases and are primarily located in the vacuole (Wang *et al*., 2018). VPEs are essential for the maturation of various vacuolar proteins and can be responsible for the degradation of recombinant proteins in plants (Outchkourov *et al*., 2003; Pillay *et al*., 2016). Although PLCPs and VPEs are two distinct types of Cys proteases, their activity can both be inhibited by cystatins (Martinez *et al*., 2007). We performed ABPP on leaf extracts to investigate if VPEs are inhibited by *Sl*CYS8. The fluorescent JOPD1 probe was used to monitor VPE activity upon *Sl*CYS8 expression as it carries an Asp residue at the P1 position and a Pro residue at the P2 position that prevents the labelling of PLCPs (Lu *et al*., 2015). Labelling by JOPD1 was unchanged upon *Sl*CYS8 expression, except for minor increases at ~40 kDa in *Sl*CYS8‐expressing leaves that might be the result of a stabilizing effect initiated by the depletion of most PLCPs (Figure 2b). As many Ser hydrolases also accumulate intracellularly, ABPP was performed on total leaf extracts using (FP)‐TAMRA probe. Activity profiles of Ser hydrolases were unaffected by *Sl*CYS8 expression or the inactive ^Q47P^ *Sl*CYS8 mutant (Figure 2c). Taken together, these results suggest that *Sl*CYS8 inhibits exclusively PLCPs.

### *In vitro* assays confirm direct inhibition of PLCPs by *Sl*CYS8

**Figure 3:**
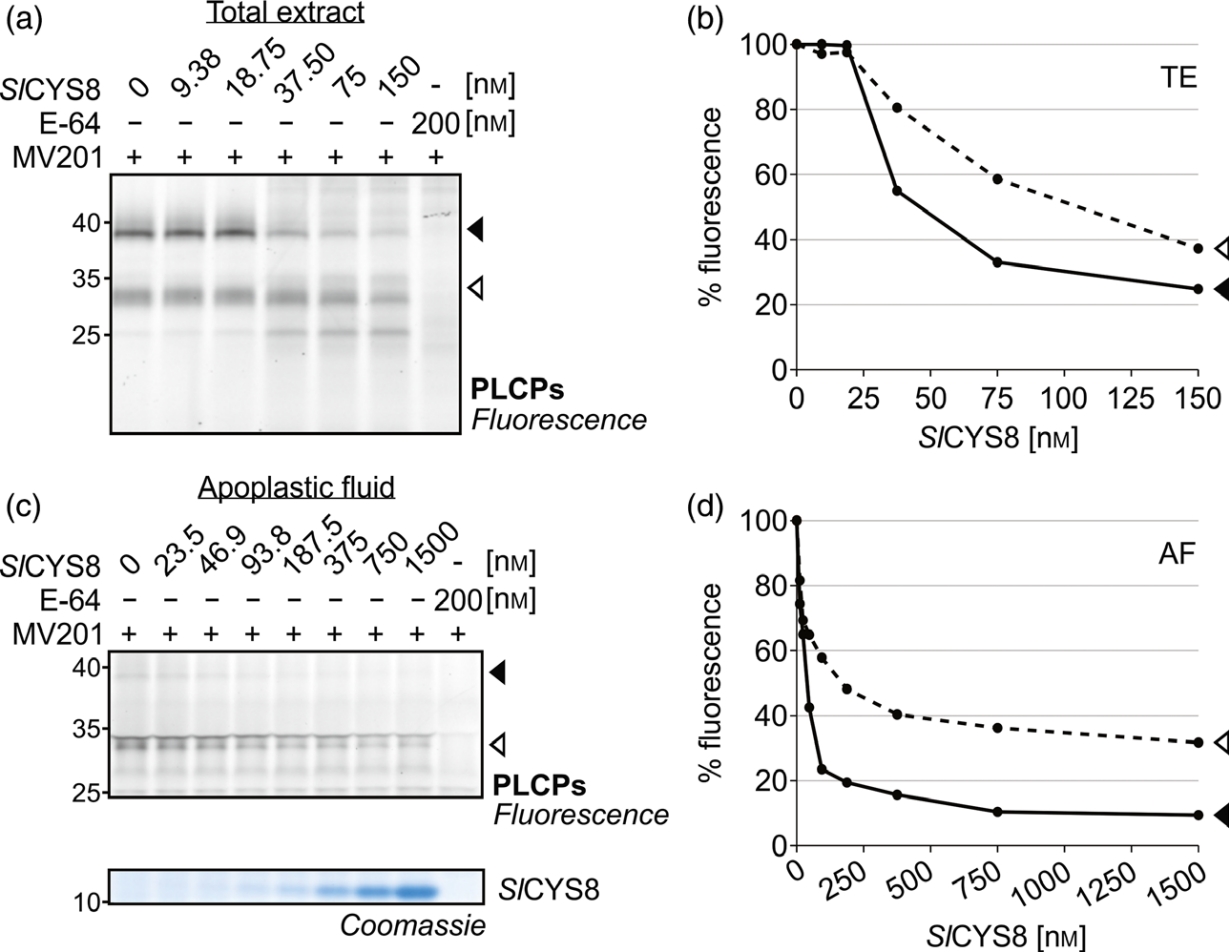
Purified *Sl* CYS8 suppresses labelling of PLCPs in total leaf extracts and apoplastic fluids. (a) Total extracts (TE) and (c) apoplastic fluids (AF) from plants agroinfiltrated with an empty vector control were isolated and pre‐incubated with various concentrations of *Sl* CYS8 purified from *E. coli*, prior to PLCP labelling with MV201. Quantification of fluorescent band intensity (b and d) shows that PLCP activity is dependent on *Sl* CYS8 concentration. As a control, plant extracts were mixed and pre‐incubated with E‐64 inhibitor before MV201 labelling. A Coomassie blue‐stained gel shows *Sl* CYS8 in the samples.

To test if the impact of *Sl*CYS8 on PLCP activity is direct, total leaf extracts from agroinfiltrated plants were pre‐incubated with 0–150 nm purified *Sl*CYS8 produced in *Escherichia coli*, prior to labelling with MV201. Addition of purified *Sl*CYS8 suppressed the two major PLCP signals (Figure 3a), similar to what was observed *in vivo* (Figure 2a). Fluorescence intensities were quantified and plotted against *Sl*CYS8 concentration, showing that activity depletion is dependent on the *Sl*CYS8 concentration (Figure 3b). Inhibition assays were also performed on apoplastic fluids isolated from agroinfiltrated plants pre‐incubated with 0–1500 nm purified *Sl*CYS8 (Figure 3c). The data showed a similar concentration‐dependent inhibition of PLCPs in apoplastic extracts, reaching 90% and 65% inhibition of the major ~38 and ~33 kDa signals, respectively (Figure 3d). These results confirm a direct inhibitory effect of *Sl*CYS8 on endogenous *N. benthamiana* PLCPs.

### Activity‐based proteomics reveals *N. benthamiana* PLCPs inhibited by *Sl*CYS8

**Figure 4:**
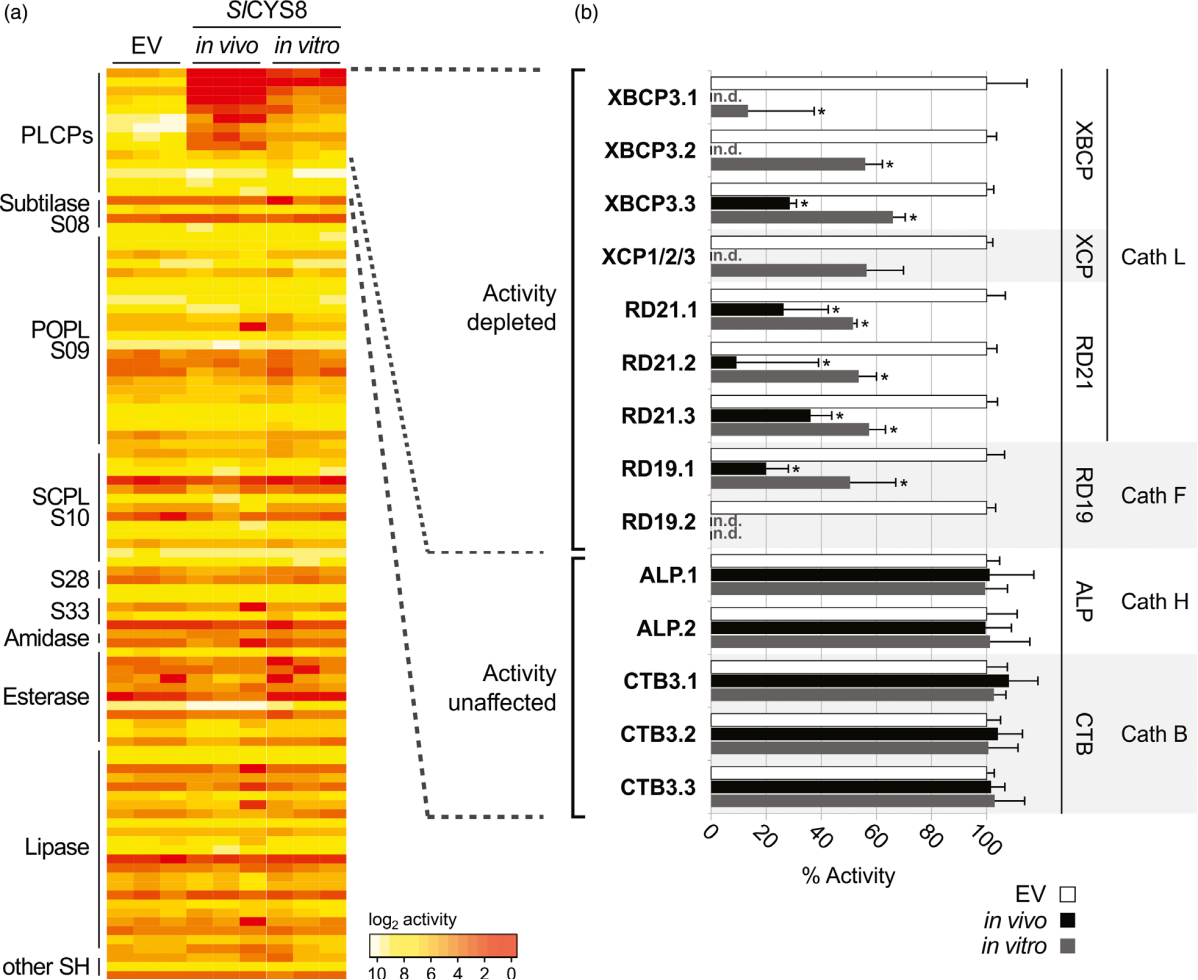
Activity‐based proteomics reveals nine endogenous PLCPs that are inhibited by *Sl* CYS8. Total leaf extracts were isolated from plants expressing *Sl* CYS8 (*in vivo*) or expressing an empty vector (EV), pre‐incubated with or without 280 nm of purified *Sl* CYS8 (*in vitro*). Proteins were labelled with DCG04 and FP‐biotin to label PLCPs and Ser hydrolases, respectively. Labelled proteins were purified and identified by MS. (a) Heatmap showing active enzymes that were significantly enriched (FDR < 0.05) compared to a no‐probe control, fold inhibition serves as a proxy for activity. Most PLCP activities were depleted upon *Sl* CYS8 expression, while no Ser hydrolase was inhibited. (b) Identified PLCPs were grouped by subfamilies and the activity was normalized to the EV control (100%). Bars are the means of three biological replicates ± SE. Star indicates significant differences from the EV control (Student's *t* test, *P *<* *0.05). PLCP subfamilies: Xylem and bark Cys proteases (XBCPs), Xylem‐specific Cys proteases (XCPs), Resistant‐to‐Desiccation‐21 proteases (RD21s), Resistant‐to‐Desiccation‐19 proteases (RD19s), Aleurain‐Like proteases (ALPs) and Cathepsin‐B‐like proteases (CTBs). n.d.; not detected. MS dataset is presented in Table S1.

To identify the proteases that are inhibited by *Sl*CYS8, we performed activity‐based proteomics (ABPP‐MS) on leaf extracts. Total extracts from agroinfiltrated leaves expressing *Sl*CYS8 (*in vivo*) or empty vector control were pre‐incubated with or without 280 nm of purified *Sl*CYS8 and labelled with a mix of two biotinylated probes to label both PLCPs and Ser hydrolases (Greenbaum *et al*., 2000; Liu *et al*., 1999). Labelled proteins were captured on avidin beads, digested with trypsin and quantified by mass spectrometry (MS). 14 PLCPs and 87 Ser hydrolases were enriched at a confidence level of 95% (*P *<* *0.05) compared to the no‐probe control, indicating that they were active. No Ser hydrolase was convincingly affected by the presence of *Sl*CYS8 *in vivo* or *in vitro* (Figure 4a and Table S1), consistent with the fluorescent profiling data (Figures 1 and 2).

Nine PLCPs were significantly less labelled in leaves expressing *Sl*CYS8 when compared to the empty vector control, suggesting that their activity was depleted by *Sl*CYS8 both *in vivo* and *in vitro*. These nine inhibited PLCPs belong to four subfamilies: Xylem and bark Cys proteases (XBCPs), Xylem‐specific Cys proteases (XCPs), Resistant‐to‐Desiccation‐19 proteases (RD19s) and Resistant‐to‐Desiccation‐21 proteases (RD21s). Two XBCP‐like and one RD21‐like PLCPs were more inhibited upon *Sl*CYS8 expression *in vivo* compared to extracts treated with *Sl*CYS8 *in vitro* (Figure 4b), potentially due to incomplete inhibition of the proteases using 280 nm *Sl*CYS8. These nine inhibited PLCPs are strong candidates to be implicated in proteolytic processing of recombinant proteins in agroinfiltrated leaves.

Our data are consistent with the literature. Some of the PLCPs identified in total extracts may cause the signals in apoplastic fluids, as earlier work revealed that the extracellular space contains XBCP, XCP, RD21 and RD19 proteases (Grosse‐Holz *et al*., 2018a). A recent study showed that purified RD21‐like NbCYSP6 and XCP‐like NbCYSP7 proteases rapidly degrade antibodies *in vitro* (Paireder *et al*., 2017). Silencing the gene encoding a RD21‐like protease (NtCYSP6) in tobacco also significantly enhanced accumulation of recombinant proteins (Duwadi *et al*., 2015).

In contrast, labelling of Aleurain‐like proteases (ALPs) and Cathepsin‐B‐like proteases (CTBs) was unaffected either by *Sl*CYS8 expressed in plants (*in vivo*) or purified *Sl*CYS8 (*in vitro*; Figure 4b). This is consistent with ALPs being cathepsin‐H‐like aminopeptidase rather than endopeptidase, as one side of the substrate‐binding groove is occupied by a minichain (Gunčar *et al*., 1998; Holwerda and Rogers, 1992). The shorter substrate‐binding groove of ALPs may not accommodate the tripartite wedge of *Sl*CYS8. The ALP subfamily might have a minor contribution in proteolytic processing of recombinant proteins, as previous *in vitro* assays showed negligible effect of NbALP on antibody stability (Niemer *et al*., 2016). CTBs are peptidyl‐peptidases with an occluding loop that blocks part of the substrate‐binding groove (Musil *et al*., 1991; Tsuji *et al*., 2008). The occluding loop may prevent its inhibition by *Sl*CYS8. Thus, even though NbCathB protease, a sequence 93% identical to the identified NbCTB3.3 protease, can degrade antibodies *in vitro* (Niemer *et al*., 2016), these CTBs probably do not contribute to recombinant protein degradation. Taken together, these data confirm overall the impact of *Sl*CYS8 expression on endogenous proteases and indicate that XBCP, XCP, RD21 and RD19‐like proteases may constitute the core of the recombinant protein degradation machinery that is blocked by *Sl*CYS8.

### *Sl*CYS8 inhibits different isoforms of granulin‐containing proteases

**Figure 5:**
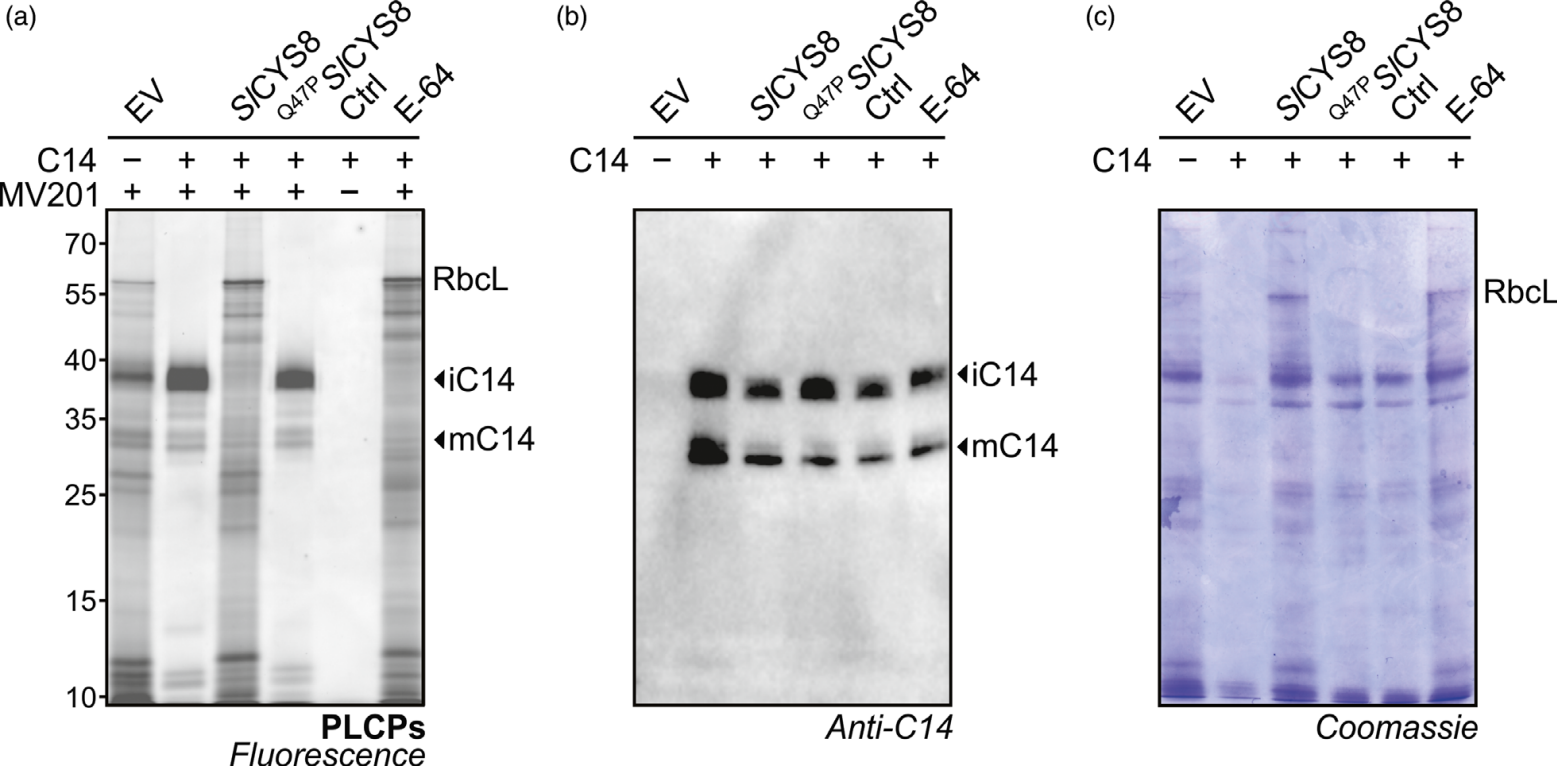
*Sl* CYS8 inhibits different isoforms of granulin‐containing proteases. (a) Leaf extracts from agroinfiltrated plants overexpressing C14, the tomato RD21‐like protease, with or without *Sl* CYS8 were labelled with MV201 to monitor PLCP activity. In‐gel fluorescence confirmed inhibition of C14 upon *Sl* CYS8 co‐expression, while no effect was observed when co‐expressed with the inactive ^Q47^ ^P^ *S* *l* CYS8 mutant. As a control, plant extracts were mixed and pre‐incubated with E‐64 inhibitor before incubation with or without (Ctrl) the probe. (b) Immunoblot shows equal amount of the intermediate protease (iC14) and the mature (mC14) form of C14 protease. (c) Coomassie gel shows that protein degradation occurred upon C14 expression and is prevented by co‐expressing *Sl* CYS8. The large subunit of ribulose‐1,5‐bisphosphate carboxylase/oxygenase (RbcL) is stabilized in *Sl* CYS8‐expressing leaves.

To confirm that *Sl*CYS8 inhibits both the intermediate and the mature forms of granulin‐containing proteases, we tested C14, the tomato orthologue of the *N. benthamiana* RD21.1 (Kaschani *et al*., 2010; Shabab *et al*., 2008). Activity‐based profiling of leaves transiently expressing C14 displayed a strong ~38 kDa signal of the intermediate form (iC14) carrying the granulin domain and a weaker signal at ~33 kDa corresponding to the mature protein (mC14) with no granulin domain (Figure 5a). Co‐expressing *Sl*CYS8 significantly suppressed the activity of both active C14 isoforms and endogenous NbRD21‐like proteases, while not affecting C14 accumulation in leaves (Figure 5a and b). A Coomassie gel showed that protein degradation occurs faster upon C14 expression and co‐expression of C14 with *Sl*CYS8 prevented this degradation (Figure 5c). Staining protein gels is a simple assay to monitor proteolytic activity of RD21‐like proteases during extraction (Gu *et al*., 2012). These results underline the impact of *Sl*CYS8 on protease isoforms and confirm the broad inhibition of RD21‐like proteases by *Sl*CYS8.

## Conclusion

Proteolytic degradation of recombinant proteins by endogenous plant proteases is a major limiting factor in molecular pharming. Different strategies have been used to suppress unwanted proteolysis *in planta* (Mandal *et al*., 2016), but the responsible proteases have not yet been identified. The tomato cystatin *Sl*CYS8 is a stabilizing co‐expression partner or a fusion partner for proteins targeted to the plant cell secretory pathway (Grosse‐Holz *et al*., 2018b; Jutras *et al*., 2016; Sainsbury *et al*., 2013). In this study, we used *Sl*CYS8 to identify target proteases that may contribute to low yields of foreign recombinant proteins in *N. benthamiana*. Our data highlight that *Sl*CYS8 specifically inhibits PLCPs, inside and outside the cells, with no impact on the activity of vacuolar‐processing enzymes and Ser hydrolases. Proteomics revealed nine inhibited PLCPs and protein gels confirm that *Sl*CYS8 inhibits different isoforms of an RD21‐like protease, preventing protein degradation. We thus discovered unique proteases in agroinfiltrated leaves that are promising targets to knockout. Depletion of these PLCPs by genome editing could reduce protein proteolysis in the secretory pathway of *N. benthamiana*.

## Experimental procedures

### Plasmid constructs

Gene expression constructs for secreted *Sl*CYS8 (GenBank Accession No. AF198390) and its inactive variant ^Q47P^ *Sl*CYS8 were described previously (Sainsbury *et al*., 2013). An ‘empty’ vector (bearing the coding sequence of gene silencing suppressor P19) was used as a negative control. The vectors were transformed into *Agrobacterium tumefaciens* strain LBA4404 and used for agroinfiltration. Gene expression construct for secreted tomato C14 was described previously (Kaschani *et al*., 2010). All gene constructs were proof checked by automatic DNA sequencing.

### Transient expression in leaves

*Nicotiana benthamiana* plants were kept in a growth room at 21 °C under a 16/8 h light/dark regime. Agrobacteria containing the binary expression plasmids were grown for 21 h at 28 °C with agitation in LB containing appropriate antibiotics. Bacteria were collected by centrifugation at 2000 ***g*** for 5 min at room temperature (RT), resuspended in infiltration buffer (10 mm 2‐(N‐morpholino) ethanesulfone (MES), 10 mm MgCl_2_, pH 5.7, 100 μm acetosyringone) to OD_600_ = 0.5 and left for 2 h at 28 °C with agitation to recover. All bacterial suspensions were mixed in a 1 : 1 ratio with a suspension of *A. tumefaciens* harbouring an expression cassette for the silencing suppressor protein P19 of artichoke mottled crinkle virus (Lombardi *et al*., 2009). The first and second fully expanded leaves of pre‐flowering *N. benthamiana* (4–5 weeks old) were infiltrated with the bacteria suspensions using a needleless syringe (D'Aoust *et al*., 2009). Leaf tissue was harvested 6 days post‐infiltration (dpi) to allow for maximum protein accumulation in the presence of the P19 silencing suppressor. Three independent replicates including leaves of three plants were used for each treatment to minimize variation of protein expression levels and to allow for statistical analysis of the data**.**

### Protein extraction

For total leaf extracts, infiltrated leaf tissue was harvested as leaf discs, flash‐frozen in liquid nitrogen and pulverized using a pestle and a mortar. Proteins were extracted in three volumes (v/fresh weight) of cold 50 mm sodium acetate (NaAc), pH 5, containing 5 mm DTT, and centrifuged at 16 000 ***g*** for 20 min at 4 °C. For apoplastic fluid extraction, six *N. benthamiana* leaves per sample were detached and vacuum‐infiltrated with ice‐cold water, dried on the surface and placed in a needle/plunger‐less syringe inserted into a 50 mL Falcon tube. Apoplastic fluids were collected by centrifugation at 2000 ***g***, 4 °C for 25 min and used immediately for protein analysis.

### Activity‐based protein profiling

Forty‐eight μL of leaf extract or apoplastic fluid adjusted to 50 mm NaAc, pH 5, 5 mm DTT, was pre‐incubated with or without 0.2 mm of inhibitor (E‐64; DCI, 3,4‐Dichloroisocoumarin; YVAD, Ac‐Tyr‐Val‐Ala‐Asp‐Chloromethylketone) for 30 min, and then incubated for 3.5 h at room temperature with fluorescent probes targeting papain‐like cysteine proteases (MV201; Richau *et al*., 2012) or vacuolar‐processing enzymes (JOPD1; Lu *et al*., 2015) or incubated for 1 h with an FP‐TAMRA probe targeting Ser hydrolase activity. FP‐TAMRA was obtained from Thermo (88318); MV201 and JOPD1 were described before (Lu *et al*., 2015; Richau *et al*., 2012). ABPP reactions were ended by adding 1 mL cold acetone. Samples were centrifuged for 3 min at 13 000 rpm and the supernatant was discarded. Proteins were resuspended in gel loading buffer (100 mm Tris‐HCl, pH 6.8, 200 mm DTT, 4% w/v SDS, 0.02% w/v bromophenol blue, 25% v/v glycerol), heated for 5 min at 95 °C, and resolved by 12% w/v SDS‐PAGE in reducing conditions. SDS‐PAGE gels were scanned on a Typhoon scanner (Amersham/GE Healthcare, Little Chalfont, UK) using Cy3 settings to detect in‐gel fluorescence. Image data were analysed using the Open source software ImageJ (http://rsb.info.nih.gov/ij/) to quantify band fluorescence intensity. Background values were subtracted from each image based on the average value of images acquired from no‐probe controls. Bands from three biological replicates were used in each case.

### Immunoblotting

Proteins resolved by 12% w/v SDS‐PAGE gel were electrotransferred onto a PVDF membrane using the TransBlot Turbo system (Bio‐rad, Hercules, CA)*. Sl*CYS8 was detected using custom‐made primary IgG raised in rabbit against anti‐*Sl*CYS8 (Robert *et al*., 2013; Agrisera, Sweden, 1/5000) and goat anti‐rabbit horseradish peroxidase (HRP)‐conjugated secondary antibodies (Agrisera, Sweden, 1/10 000). C14 was detected with anti‐C14 polyclonal IgG (1/1000) raised in rabbit (Kaschani *et al*., 2010) and goat anti‐rabbit HRP secondary antibodies (Agrisera, Sweden, 1/5000). Non‐specific binding sites on PVDF membranes were blocked for 1 h with 5% w/v skimmed milk powder in PBS buffer containing 0.025% v/v Tween‐20, which also served as antibody dilution buffer. Chemiluminescent signals were detected using the Clarity ECL Western blotting detection kit (Bio‐rad), and protein–antibody complex signals were captured on a Gel Doc imager (Bio‐rad).

### Bacterial expression of recombinant *Sl*CYS8

Glutathione S‐transferase (GST)‐tagged *Sl*CYS8 expression in *E. coli* was carried out as reported (Goulet *et al*., 2008). Briefly, 5 mL pre‐cultures of BL21 *E. coli* cells were incubated overnight in LB medium at 37 °C, with carbenicillin added in the culture medium. The pre‐cultures were transferred into 250 mL LB cultures and incubated at 37 °C until an OD_600_ of 0.6. Protein expression was induced by the addition of 0.5 mm of isopropyl β‐D‐1‐thiogalactopyranoside (IPTG) and the cultures were incubated for another 6 h at 30 °C. Bacteria were collected by centrifugation at 3000 ***g*** for 10 min, submitted to four freeze‐thaw cycles, resuspended in 3 mL of lysis buffer (50 mm Tris pH 8.0, 5% m/v sucrose, 50 mm EDTA, 5% v/v Triton X‐100, 1 mm PMSF), incubated on ice for 5 min and centrifuged at 13 000 ***g*** for 10 min 4 °C. The supernatant was collected and incubated with 250 μL of Glutathione Sepharose‐4B beads (GE Healthcare Life Sciences, Chicago, IL) for 60 min with low agitation. Samples were centrifuged 5 min at 500 g to spin down the beads and the supernatant was discarded. Beads were washed three times using 5 mL of 50 mm Tris pH 8.0 and centrifuged 5 min at 500 g. The last wash was with 5 mL of Factor X_a_ cleavage buffer (20 mm Tris‐HCl, 100 mm NaCl, 2 mm CaCl_2_, pH 8.0). After centrifugation (5 min at 500 ***g***), the supernatant was discarded and 300 μL of Factor X_a_ cleavage buffer was added to the beads. The GST fusion partner was removed by adding 5 μL of Factor X_a_ enzyme (NEB, Ipswich, MA). The samples were incubated with Factor X_a_ for 16 h at room temperature with gentle agitation, and centrifuged at 6000 ***g*** for 5 min. Proteins in the supernatant were assayed using the standard Bradford protocol and maintained at −80 °C until further use.

### *In vitro* inhibition assays

Total leaf extracts (TE) or apoplastic fluids (AF) from leaves agroinfiltrated with the empty vector control were extracted in sodium acetate buffer and purified *Sl*CYS8 protein variants were added at different concentrations (0–150 nm for TE and 0–1500 nm for AF) for a 30‐min incubation at room temperature prior to PLCP labelling as previously described. The same amount of factor X_a_ cleavage buffer was added in the no *Sl*CYS8 control. Inhibition rates were determined by the quantification of band fluorescence intensity compared to the no *Sl*CYS8 control. Incremental amounts of purified *Sl*CYS8 added in the samples were visualized on Coomassie blue‐stained polyacrylamide gels following 12% SDS‐PAGE.

### Activity‐based proteomics

Leaves from different plants were infiltrated with agrobacterial suspensions (OD_600_ = 0.5) harbouring the P19 vector or 1:1 (v/v) *Sl*CYS8:P19 vectors. Proteins were extracted at 6 dpi as described above and diluted to 1.5 mg/mL. In the ‘*Sl*CYS8 *in vitro*’ samples, 3 ng of purified *Sl*CYS8 from *E. coli* were added per 1 μL volume. All samples were incubated at RT on a rotator for 45 min. An amount of 1 mL of each sample was then incubated with 5 μm FP‐biotin (Sigma 88317) and 5 μm DCG04 (Greenbaum *et al*., 2000) for 5 h on a rotator at RT in darkness. A no‐probe control contained a mix of equal volumes of each sample and DMSO. Samples were transferred to 15 mL falcon tubes and 4 mL ice‐cold methanol, 1 mL ice‐cold chloroform and 3 mL ice‐cold water were added subsequently, vortexing the sample after each step. Samples were centrifuged for 30 min at 3000 ***g*** (4 °C). The upper aqueous layer was carefully removed, 4 mL of methanol was added and the samples were centrifuged for 30 min at 3000 ***g*** (4 °C). The supernatant was discarded and the pellet was resuspended in 2 mL PBS containing 1.2% w/v sodium dodecyl sulphate (SDS) and then diluted using 5 mL PBS. Proteins were denatured by heating at 90 °C for 8 min and cooled on ice, before dilution with 3 mL of PBS and stored at −20 °C until the next day. After thawing, 130 μL avidin beads (Sigma A9207, pre‐washed three times in 1X PBS) was added to each sample. Samples were incubated on a rotator for 1 h at room temperature and centrifuged for 7 min at 400 *g*. The supernatant was discarded and the beads were washed four times in 1% w/v SDS and twice in water after centrifuging for 7 min at 400 *g* each time. Purified proteins on the beads were reduced in 256 μL 50 mm Tris‐HCl pH 8.0, 8 m urea, 10 mm DTT for 15 min at 65 °C with agitation in darkness, then cooled to 35 °C and alkylated by adding 12.5 μL of 400 mm iodoacetamide in 50 mm Tris‐HCl pH 8.0 and incubating for 30 min with agitation in darkness. An amount of 4 μL of Trypsin‐LysC (Promega V5071) was added to each sample and the LysC digest was performed for 3 h at 37 °C under gentle agitation. Samples were then diluted in 50 mm Tris‐HCl pH 8.0 to reach a final urea concentration of less than 1 m and trypsin digestion was performed for 16 h at 37 °C with agitation. The samples were centrifuged for 3 min at 1000 ***g***, and 0.1% v/v trifluoracetic acid added to the supernatant in new tubes. The peptides were purified using Sep‐Pak C18 cartridges (Waters, 610 Centennial Park, Herts, UK) according to the manufacturer's instructions.

### Mass spectrometry

Mass spectrometry analysis is described in Appendix S1.

### Bioinformatics tools for leaf proteome analysis

Peptide spectra were annotated using Andromeda (Cox *et al*., 2011). Included modifications were carbamidomethylation (static) and oxidation, N‐terminal acetylation and carbamylation of Lysines and N‐termini (dynamic). Protein quantification was performed using MaxQuant version 1.5.5.30 (Tyanova *et al*., 2016a), including all modifications. Filtering and imputation of missing values using default settings were performed in Perseus (Tyanova *et al*., 2016b) and further data analysis carried out in R using the data.table and ggplot packages (Ihaka and Gentleman, 1996; Wickham, 2016). Sequence analyses and plasmid design were performed in Geneious (Kearse *et al*., 2012).

## Conflicts of interest

The authors declare no conflicts of interest.

## Supporting information


**Table S1** Proteases identified by ABPP‐MS.

**Appendix S1** Mass spectrometry analysis.
