# Supplementary material for: Activity‐based proteomics reveals nine target proteases for the recombinant protein‐stabilizing inhibitor Sl CYS8 in Nicotiana benthamiana
Source: Plant Biotechnol J. 2019 Mar 14;17(8):1670–8. doi: 10.1111/pbi.13092 (PMC6662110; doi:10.1111/pbi.13092)
Supplement: Supplementary file 2 — Appendix S1 Mass spectrometry analysis. [file PBI-17-1670-s002.docx]

# Supplementary Method S1 – Mass spectrometry

**LC-MS/MS.**

Acidified tryptic digests were desalted on home-made two disc C18 StageTips as previously described (Rappsilber et al., 2007). After elution, samples were dried using a vacuum concentrator (Eppendorf) and peptides were recovered in 10 µL 0.1 % formic acid solution. Experiments were performed on an Orbitrap Elite instrument (Michalski et al. 2012) coupled to an EASY-nLC 1000 liquid chromatography (LC) system (Thermo). The analytical column was a fused silica capillary (75 µm × 35 cm) with an integrated PicoFrit emitter (New Objective) packed in-house with Reprosil-Pur 120 C18-AQ 1.9 µm resin. The column was encased by a column oven (Sonation) and attached to a nanospray flex ion source (Thermo). The temperature was adjusted to 45 °C during data acquisition. The LC was equipped with two mobile phases: solvent A (0.1% formic acid in water) and solvent B (0.1% formic acid in acetonitrile, ACN). Peptides were directly loaded onto the analytical column with a maximum flow rate that would not exceed the set pressure limit of 980 bar (usually around 0.6 – 1.0 µL/min). Peptides were subsequently separated on the analytical column by running a 140 min gradient of solvent A and solvent B (start with 7% B; gradient 7% to 35% B for 120 min; gradient 35% to 100% B for 10 min and 100% B for 10 min) at a flow rate of 0.3 µl/min. The mass spectrometer was operated using Xcalibur software (version 2.2 SP1.48) and was set in the positive ion mode. Precursor ion scanning was performed in the Orbitrap analyzer (FTMS; Fourier Transform Mass Spectrometry) in the scan range of *m/z* 300-1800 and at a resolution of 60000 with the internal lock mass option turned on (lock mass was 445.120025 *m/z*, polysiloxane) (Olsen et al. 2005). Product ion spectra were recorded in a data dependent fashion in the ion trap (ITMS) in a variable scan range and at a rapid scan rate. The ionization potential (spray voltage) was set to 1.8 kV. Peptides were analyzed using a repeating cycle consisting of a full precursor ion scan (3.0 × 10^6^ ions or 50 ms) followed by 12 or 15 product ion scans (1.0 × 10^4^ ions or 50 ms). Peptides were isolated based on their intensity in the full survey scan (threshold of 500 counts) for tandem mass spectrum (MS2) generation. Collision induced dissociation (CID) energy was set to 35% for the generation of MS2 spectra. During MS2 data acquisition dynamic ion exclusion was set to 120 seconds with a maximum list of excluded ions consisting of 500 members and a repeat count of one. Ion injection time prediction, monoisotopic precursor selection and charge state screening were enabled. Only charge states higher than one were considered for fragmentation.

**Peptide and Protein Identification using MaxQuant.**

RAW spectra were submitted to an Andromeda search in MaxQuant (1.5.3.30) using the default settings (Cox et al., 2011; 2008). Label-free quantification (LFQ) and match-between-runs was activated (Cox et al., 2014). The MS/MS spectra data were searched against an in-house generated *Nicotiana benthamiana* (taxonomy-id: 4100) database (DB05_representative_proteins_curated.fasta; 74091 entries) (Grosse-Holz et al., 2018). All analysis included a contaminant database search (as implemented in MaxQuant) to estimate the level of contamination. Andromeda searches allowed oxidation of methionine residues (16 Da) and acetylation of the protein *N*-terminus (42 Da) as dynamic modifications and the static modification of cysteine (57 Da, alkylation with iodoacetamide). Enzyme specificity was set to “Trypsin/P” with two missed cleavages allowed. Andromeda was set to Orbitrap and the precursor mass tolerance was set to ±20 ppm (first search) and ±4.5 ppm (main search). The MS/MS match tolerance was set to ±0.5 Da. False Discovery Rates (FDR) were set to 0.01 (based on target-decoy approach). Minimum peptide length was seven amino acids. For protein quantification, modified peptides and razor peptides were allowed for quantification. The minimum score for modified peptides was 40. Label-free quantification (LFQ) was switched on and peptides were considered for quantification with a minimum ratio count of two. Retention times were recalibrated based on the built-in nonlinear time-rescaling algorithm. MS/MS identifications were transferred between LC-MS/MS runs with the “match between runs” option in which the maximal match time window was set to 0.7 min and the alignment time window set to 20 min. The quantification is based on the “value at maximum” of the extracted ion current. At least two quantitation events were required to quantify a protein. Further analysis and filtering of the results was done in Perseus v1.5.5.3 (Tyanova et al., 2016). Briefly, only protein groups with at least two identified unique peptides over all runs were considered for further analysis. For quantification, we combined related biological replicates to categorical groups and investigated only proteins that were found in at least one categorical group in all three biological replicates. Comparison of protein group quantities (relative quantification) between different MS runs is based solely on the LFQ’s as calculated by MaxQuant (MaxLFQ algorithm) (Cox et al., 2014).

# References

Cox J, Neuhauser N, Michalski A, Scheltema RA, Olsen JV, et al. (2011) Andromeda: a peptide search engine integrated into the MaxQuant environment. *J. Proteome Res*., **10**, 1794-1805.

Cox J, Mann M (2008) MaxQuant enables high peptide identification rates, individualized p.p.b.-range mass accuracies and proteome-wide protein quantification. *Nat. Biotechnol*., **26**, 1367-1372.

Cox J, Hein MY, Luber CA, Paron I, Nagaraj N, et al. (2014) Accurate proteome-wide label-free quantification by delayed normalization and maximal peptide ratio extraction, termed MaxLFQ. *Mol. Cell. Proteomics,* **13**, 2513-2526.

Grosse-Holz F, Kelly S, Blaskowski S, Kaschani F, Kaiser M, et al. (2018) The transcriptome, extracellular proteome and active secretome of agroinfiltrated *Nicotiana benthamiana* uncover a large, diverse protease repertoire. *Plant Biotechnol. J.*, **16**, 1068–1084.

Michalski A, Damoc E, Lange O, Denisov E, Nolting D, et al. (2012) Ultra high resolution linear ion trap Orbitrap mass spectrometer (Orbitrap Elite) facilitates top down LC MS/MS and versatile peptide fragmentation modes. *Mol. Cell. Proteomics,* **11**, O111 013698.

Olsen JV, de Godoy LM, Li G, Macek B, Mortensen P, et al. (2005) Parts per million mass accuracy on an Orbitrap mass spectrometer via lock mass injection into a C-trap. *Mol. Cell. Proteomics,* **4**, 2010-2021.

Rappsilber J, Mann M, Ishihama Y (2007) Protocol for micro-purification, enrichment, pre-fractionation and storage of peptides for proteomics using StageTips*. Nat. Protoc*., **2**, 1896-1906.

Tyanova S, Temu T, Sinitcyn P, Carlson A, Hein MY, et al. (2016) The Perseus computational platform for comprehensive analysis of (prote)omics data. *Nat. Methods,* **13**, 731-740.
